# A New Look at Infant Problem-Solving: Using DeepLabCut to Investigate Exploratory Problem-Solving Approaches

**DOI:** 10.3389/fpsyg.2021.705108

**Published:** 2021-11-08

**Authors:** Hannah Solby, Mia Radovanovic, Jessica A. Sommerville

**Affiliations:** Department of Psychology, University of Toronto, Toronto, ON, Canada

**Keywords:** cognitive development, exploration, infant development, motion capture technology, automated behavioral analysis, problem solving, DeepLabCut

## Abstract

When confronted with novel problems, problem-solvers must decide whether to copy a modeled solution or to explore their own unique solutions. While past work has established that infants can learn to solve problems both through their own exploration and through imitation, little work has explored the factors that influence which of these approaches infants select to solve a given problem. Moreover, past work has treated imitation and exploration as qualitatively distinct, although these two possibilities may exist along a continuum. Here, we apply a program novel to developmental psychology (DeepLabCut) to archival data (Lucca et al., 2020) to investigate the influence of the effort and success of an adult’s modeled solution, and infants’ firsthand experience with failure, on infants’ imitative versus exploratory problem-solving approaches. Our results reveal that tendencies toward exploration are relatively immune to the information from the adult model, but that exploration generally increased in response to firsthand experience with failure. In addition, we found that increases in maximum force and decreases in trying time were associated with greater exploration, and that exploration subsequently predicted problem-solving success on a new iteration of the task. Thus, our results demonstrate that infants increase exploration in response to failure and that exploration may operate in a larger motivational framework with force, trying time, and expectations of task success.

## Introduction

The ability to overcome obstacles to achieve one’s goals is crucial to success across a broad range of contexts. Problem-solving is particularly ubiquitous early in life. Infants are faced with a multitude of new problems every day such as obtaining desirable, out-of-reach objects, navigating around barriers, and learning to operate new toys. Research suggests that infants typically adopt one of two approaches to solving problems: infants imitate the problem-solving solutions of others (e.g., Provasi et al., 2001; Esseily et al., 2010) or explore to generate their own solutions (e.g., Willatts, 1999; Fagard et al., 2014). However, the circumstances that influence whether infants adopt problem-solving approaches modeled for them versus explore new approaches are not well understood. In this paper, we investigate whether the nature of the social input infants receive (namely, the effort and success of an adult’s modeled problem-solving solution) and infants’ own firsthand experience influence the degree to which infants use imitative versus exploratory problem-solving solutions.

Infants are capable learners and independently generate new solutions to problems via exploration. A variety of work suggests that when infants are presented with problem-solving paradigms in which they cannot obtain goal objects directly, they implement novel solutions (Goldfield, 1983; Willatts and Rosie, 1988; Babik et al., 2018). For instance, by the end of the first year of life, infants discover that they must reach or crawl around a barrier to retrieve a toy (Lockman, 1984; Lockman and Adams, 2001), and they can pull a cloth supporting an out-of-reach toy to get the toy (Sommerville and Woodward, 2005a,b). Critically, infants not only implement known solutions, but often explore new solutions iteratively until success is achieved (Willatts, 1990). With age, infants’ ability to explore and innovate novel problem-solving solutions continues to improve. By 16 months of age, infants discover that they can use a rake as a tool to bring an out-of-reach toy into reach (Fagard et al., 2014). Thus, from an early age infants engage in exploration when presented with novel problems, often leading to problem-solving success.

Simultaneously, a variety of evidence suggests that infants also rely on imitation to solve problems. By 12 months of age, infants can already solve simple problems by replicating modeled solutions across a variety of contexts (Provasi et al., 2001; Brugger et al., 2007; Fagard and Lockman, 2009; Fagard et al., 2016). For example, 1-year-olds will learn from an adult model to grasp one end of a box while simultaneously raising a lid to overcome suction, to orient a bottle upside-down to retrieve a wooden peg, and to use a stick as a tool to retrieve a toy from a box (Esseily et al., 2010). Thus, infants readily imitate modeled solutions to facilitate their own success on novel problems.

While there is ample evidence that infants imitate, they do not do so indiscriminately; rather, infants remove superfluous components of modeled problem-solving solutions and explore their own solutions when modeled solutions are inefficient. For example, infants only replicate the exact actions of an adult model when they are the most efficient means to achieving a goal (e.g., Schwier et al., 2006). When steps are not causally necessary, infants are likely to skip these steps (e.g., Hauf et al., 2004; Brugger et al., 2007; Schulz et al., 2008). Not only do infants deviate from imitation by omitting superfluous steps, but infants also explore alternate solutions. When infants were shown a demonstration in which an experimenter turned on a light with their head with unconstrained hands, infants often utilized their own hands to turn on the light, achieving the goal more directly (Gergely et al., 2002; Zmyj et al., 2009). These studies demonstrate that there is some degree of fluidity in terms of whether infants will imitate versus explore when solving a problem.

In studies to date, imitation is often considered as qualitatively distinct from exploration, and consequently, researchers sometimes focus selectively on one approach or the other. For instance, Brugger et al. (2007) studied the effects of step necessity and adult modeling on imitation by coding whether infants performed two modeled steps. In this way, the authors successfully studied imitation, but exploration of novel solutions was not considered. Likewise, many studies separately measure imitation and exploration by providing distinct definitions of each. For example, Muentener et al. (2018) investigated both exploration and imitation but devised separate tasks and scales to quantify each approach independently. At a global level, it is reasonable to consider the constructs of imitation and exploration separately given that researchers are trying to capture qualitatively distinct strategies. However, individuals often also engage in more nuanced explorations that involve variations on modeled solutions. For example, imagine an observer watches a model insert a key into a lock and turn it twice counterclockwise. After watching, the observer puts their own key into the lock, but it does not open. The observer may persevere in trying to reproduce the exact solution of the model, or they may enact a qualitatively distinct solution such as knocking on the door. On the other hand, the observer may also engage in micro-exploration: jiggling the key, varying force, or varying the angle they use to release the lock. When this variability is taken into consideration, it becomes clear that a continuous scale can be construed between faithful imitation and micro-exploration.

While micro-exploration has not been studied at the level of particular problem-solving strategies, there is evidence of micro-exploration within infant object interactions. Nuanced refinements of existing strategies have been argued to play a particularly important role in infancy both for acquiring motor skills (Robin et al., 1996; Keen, 2011) and for generating more complicated problem-solving strategies (Lockman, 2000). Specifically, to utilize tools and interact with objects, infants must learn to make subtle variations in their approaches, for instance by adjusting their trajectory and velocity while using a hammer (Kahrs et al., 2013) or by altering their grip on a food-laden spoon (McCarty et al., 1999), thus engaging in micro-exploration. These findings indicate that infants engage in micro-exploration to successfully handle objects and that they may also apply this ability to solve challenging problems.

Thus, the ability to integrate imitation and exploration into a continuum is important to understand the full spectrum of strategies that infants employ to solve problems. However, the ability to achieve this objective may be hampered by the inherent difficulty of quantifying imitation and exploration within a single objective and continuous scale. Research in this area has largely been accomplished through behavioral coding schemes and human raters. To do this, coders make qualitative judgments about whether a subject has imitated or explored, as well as the kind of exploratory strategy generated. In this way, participant behavior is coded and coerced into a discrete category structure. In order to investigate imitation and micro-exploration along a continuum, one must move beyond behavioral coding.

Fortunately, motion capture technology presents an avenue to generate continuous, objective measures of infants’ motor responses in order to quantify infants’ problem-solving approaches along an imitation-exploration continuum. Indeed, there is a rich history of using motion capture technology in developmental research to assess early motor, perceptual, and cognitive development (Thelen et al., 1996; Adolph et al., 2000; Berger and Adolph, 2003; Claxton et al., 2003; McCarty and Keen, 2005; Gill et al., 2009; Gottwald and Gredebäck, 2015; Jung et al., 2015; Fragaszy et al., 2016; Gottwald et al., 2017, 2019; Ingvarsdóttir and Balkenius, 2020). Advancements in artificial intelligence have expanded access to motion capture by creating free, online programs for *post hoc* analysis such as frame difference (e.g., Paxton and Dale, 2013) and computer vision methods (e.g., Ossmy et al., 2020; Cao et al., 2021). While these computer vision methods have existed for the last decade, they have not been broadly employed in the field of developmental psychology and are not yet in the toolbox of most developmental researchers.

Here, we focus particularly on DeepLabCut (DLC) which allows users to train a neural network to track motion in up to three dimensions. DLC relies on a specialized algorithm which is pre-trained (Deng et al., 2009) such that DLC’s neural network only requires a small number of frames for training and can manage lower resolution footage (Mathis et al., 2018; Cronin et al., 2019). As such, DLC is suitable for use with small samples but provides quality comparable to even commercial systems (Sturman et al., 2020; Vonstad et al., 2020). Further, DLC is incredibly versatile and is even able to track multiple, distinct individuals (Mathis et al., 2018, 2020). Thus, once trained, a researcher can utilize DLC with diverse data sets and variables of interest. Finally, the software is open-source, and its use has rapidly expanded across disciplinary lines in the last 3 years (for a scoping meta-analysis, see Table 1). The open-source nature of the program has stimulated an online community, with researchers introducing specialized packages (e.g., Fiker et al., 2020; Forys et al., 2020). As a field, developmental psychology has a history of active contribution to open-source projects, with many researchers releasing specialized packages in programming languages for others’ use (e.g., Burke, 2019; Kominsky, 2019; Sanchez et al., 2019). As such, while developmental psychologists seem largely unaware of this technology, they are well-positioned to benefit from the rich and accurate behavioral data generated by DLC, as well as to contribute to the larger DLC community.

**TABLE 1:** A breakdown of peer-reviewed studies that have used DeepLabCut, organized by field of study, between 2018 and 2021.

Field of study		Number of articles	Authors (year)
Agriculture		2	Liu et al. (2020); Fang et al. (2021)
Biology		1	Ho et al. (2021)
Biomechanics		2	Cronin et al. (2019); Cronin (2021)
Neuroscience/Neurobiology		12	Mathis et al. (2018); Wei and Kording (2018); Fiker et al. (2020); Forys et al. (2020); Fried et al. (2020); Kim et al. (2020); Mathis and Mathis (2020); Mundorf et al. (2020); Rodriguez et al. (2020); Sturman et al. (2020); Whishaw et al. (2020); Williams et al. (2020)
Orthopedics		1	White et al. (2020)
Physiology		4	Barrett et al. (2020); Haberfehlner et al. (2020); Wu et al. (2020); Brandt et al. (2021)
Psychology	Comparative Psychology	1	López Pérez et al. (2021)
	Developmental Psychology	0	
Science and Technology		5	Nath et al. (2019); Mathis et al. (2020); Vonstad et al. (2020); Huang et al. (2021); Namba et al. (2021)

*In order to identify the scope of DLC in different fields, we gathered publications that used DLC by using PsycINFO and the first 15 pages of Google Scholar with the search term “DeepLabCut.” This analysis revealed 30 peer-reviewed papers that used DLC since its inception in 2018. Of these 30 papers, zero were in the field of developmental psychology. While DLC is a state-of-the-art software and is widely acknowledged in other fields such as neuroscience (Fried et al., 2020), developmental psychology has not yet taken advantage of this software.*

The goal of the current paper was to apply DLC to archival videos (Lucca et al., 2020) to capture infants’ problem-solving approaches to a challenging problem. In the original study, Lucca et al. (2020) provided 18-month-old infants with a modeled solution to a means-end problem: infants watched an adult experimenter pull a rope that was attached to an out-of-reach transparent box containing a toy, in order to bring the box and toy within reach. Infants saw one of three demonstrations that varied in terms of effort and success. In the Easy condition, the experimenter pulled the rope, and the box immediately came within reach, allowing her to retrieve the toy. In the Hard condition, the experimenter pulled the rope five times. On the first four pulls, the box did not move despite the experimenter’s efforts. On the fifth pull, the box slowly began to move until it was completely brought into reach, allowing her to retrieve the toy. The Impossible condition demonstration was similar to the Hard condition, except that the experimenter never succeeded in moving the box and thus was unable to retrieve the toy. After observing the demonstration, infants were presented with an impossible test trial in which the toy was surreptitiously affixed to the table. This cycle was repeated three times, and the researchers measured how long infants engaged in pulling the rope to retrieve the toy, as well as negative affect, maximum pulling force, help-seeking, and hints required during a subsequent recovery trial (designed to test supported needed on a new iteration of the task).

Lucca et al. (2020) found that the effort and success of the adult model and accumulating firsthand experience with failure jointly influenced how long infants attempted to solve the problem, as well as several measures of performance. For instance, trying time dramatically decreased across trials in the Easy condition as infants experienced greater firsthand failure. Here, the success of the experimenter model suggested that infants should succeed quickly by employing the experimenter’s approach. As such, infants may have inferred that they did not have adequate skill to solve the problem. Similarly, in the Impossible condition, trying time also dramatically decreased across trials, but also started off relatively low. In this case, the experimenter’s failure, coupled with firsthand failure across trials, may have led infants to infer that the task was simply impossible. On the other hand, in the Hard condition, infants’ efforts remained relatively stable across trials. In this case, infants’ inferences about the problem were presumably influenced by both sources of information. The experimenter demonstration suggested that the problem was solvable but difficult, requiring infants to try for sufficiently long to succeed. Thus, the firsthand failure infants experienced was not surprising or demotivating, leading to continued trying despite failure. Together these findings indicated that the effort and success of the adult model, along with accumulating firsthand experience with failure, influence how long infants try to solve a given problem.

Thus, the current study had three objectives. First, we investigated how two manipulated factors, the effort and success of an adult model’s problem-solving solution and firsthand experience with problem-solving failure, influenced the degree to which infants adopt imitative versus exploratory approaches. Second, we looked at how individual differences in other performance measures on the task, such as infants’ negative affect, maximum pulling force, help-seeking, and trying time predicted infants’ exploration. Finally, we were interested in if imitative versus exploratory approaches predicted motivation on a functioning version of the task.

To address our three objectives, we trained DLC to track the coordinates of the rope handle the infants pulled during the problem-solving task. Specifically, we considered imitation in this context to have two components: (1) visible similarity to the approach employed by the experimenter, and (2) consistency of employment across time. Thus, we examined how model success and firsthand experience with failure influenced the degree of infants’ *imitative similarity* to the experimenter (i.e., the extent to which infants copied the experimenter model), as well as the *variability* in their attempted solutions (i.e., how much infants varied the location of their attempts). Imitative similarity was measured using the displacement of rope pulling in the *x-* and *y-*axes relative to imitative pulling, and variability was measured using each participant’s standard deviation of spatial displacement. Within the context of these variables, a decidedly imitative problem-solving approach would be marked by high imitative similarity and low variability, while a decidedly exploratory problem-solving approach would be marked by low imitative similarity and high variability as multiple, novel solutions would be tested.

Regarding our first research question, it is plausible that we would find a pattern similar to Lucca et al. (2020), wherein infants would respond to the effort and success of the adult model, and their firsthand failure. A successful model (Easy condition) suggests that infants should imitate the solution for similar success, consistent with prior work showing that adults and children tend to favor imitating the solutions of others when others are successful (Schulz et al., 2008; Rendell et al., 2010; Wisdom and Goldstone, 2011; Reindl and Tennie, 2018). On the other hand, when the model fails (Impossible condition), infants may be more likely to explore because they think imitation is unlikely to solve the problem. Indeed, children and adults also increase rates of exploration when modeled examples are lower quality or less reliable (Rook and van Knippenberg, 2011; Carr et al., 2015). When the adult shows it is difficult but possible to solve the problem (Hard condition), infants’ responses may fall between these two possibilities.

However, it may be the case that infants are influenced mostly by their firsthand experiences with failure, given that infants uniformly experience firsthand failure in all conditions and trials. Prior work has indicated that children increase exploration when the success of outcomes is unclear or surprising (Schulz and Bonawitz, 2007; Gweon and Schulz, 2008; Stahl and Feigenson, 2015; Bridgers et al., 2019). Thus, given infants’ consistent experience with failure during the test trials, we expected that as a group, infants would explore solutions different from the experimenter, and that greater experience with failure (within trials and across trials) would decrease imitation, as continued failure would suggest imitation was not fruitful.

In order to address the second research question, we investigated whether negative affect, maximum pulling force, help-seeking, and trying time predicted infants’ imitative versus exploratory approaches. It is possible infants’ affective responses may drive exploratory approaches, as infants may be more likely to abandon modeled solutions when frustrated. Similarly, infants may be more likely to adopt exploratory approaches when they have exerted maximal force when pulling the rope, compared to when they have only used minimal force. Addressing whether help-seeking predicted exploratory approaches will shed light on the extent to which the use of micro-exploration is predicted by the adoption of qualitatively distinct approaches. Furthermore, investigating whether trying time predicts exploratory approaches will inform whether these two metrics of performance signal conceptually related phenomena (i.e., different forms of persistence) or distinct phenomena.

Finally, we investigated whether exploratory approaches during test trials predicted hints needed during recovery trials when the task was solvable. While imitative and exploratory approaches are both means to remain engaged on the rope-pulling task, infants may generate different expectations through engagement in each approach. If a decidedly imitative approach is adopted, infants will uniformly experience failure every time they employ their method. This experience would likely lead to low expectations that the method will succeed the next time it is employed. On the other hand, each exploratory strategy infants test presents a possibility of success, even if small. Thus, infants who try a variety of strategies may require less support on a new iteration of the task.

## Materials and Methods

### Participants

Participant videos from Lucca et al. (2020) were repurposed for this study. In the original study, 96 full-term, typically developing 18-month-olds (38 females, mean age = 18.50 months, range = 17.67–19.30 months) participated. Participants had previously signed up to partake in studies through a university database and were recruited through this database for this study. Participants were parent-reported as White (*n* = 69), Asian (*n* = 3), Hispanic (*n* = 2), mixed race (*n* = 21), or declined to report (*n* = 1). The sample size was limited to that of the original study.

### Motor Skills Checklist

In order to ensure our results were not constrained by individual differences in motor coordination, a measure of gross motor development was administered. Parents were given a 24-item motor ability checklist (Loucks and Sommerville, 2013) that was a variation of the Bayley Scales of Motor Development (Bayley, 2006). Questions pertained to infant’s motor abilities and were organized in chronological order of developmental milestones (e.g., “Can your child sit alone while playing with a toy?,” “Does your child attempt to walk?,” “Can your child stand on one foot with help?”). The highest consecutive item parents checked served as a measure of motor development.

### Procedure

Lucca et al.’s (2020) procedure consisted of three components: (1) a warm-up to familiarize the infants to their new environment, (2) demonstration-test trials: the experimenter first tried to retrieve an out-of-reach toy, then the infant was given the opportunity to retrieve the out-of-reach toy (this cycle was repeated three times with three different toys), and (3) a recovery trial. A more detailed description of the methods can be found in the original publication (Lucca et al., 2020); here we highlight the most important components for the current study.

During the demonstration-test trials, caregivers were seated and wearing occluding eyeglasses while infants sat on their caregiver’s lap. In the demonstration phase, the infant observed the experimenter attempt to retrieve an out-of-reach toy in a transparent container by pulling on a rope that was attached to the container. Infants were assigned to one of three conditions. In the Easy condition, the experimenter easily retrieved the toy by pulling on the rope. In the Hard condition, the infant saw the experimenter struggle (she pulled the rope five times, and the box did not move), and eventually succeed at retrieving the toy by pulling on the rope. In the Impossible condition, the infant saw the experimenter try to pull the rope to retrieve the toy the same number of times as in the Hard condition, but she did not succeed.

During the test phase, infants were presented with the same toy in a container, attached to a rope. Unbeknownst to infants, the apparatus had been replaced with an identical looking version such that the container was stuck to the tabletop making the problem impossible to solve. Each trial ended after 120 s total had passed, or if the infant had not touched the rope for 15 s. The demonstration-test sequence was repeated three times, with three different toys. We analyzed the first 20 s of each trial for two reasons. First, as we wanted to adhere closely to the original methods. Second, as trials were variable in length and we believed it was important to have an equal amount of data from each participant for inferential purposes (i.e., data from 120 s would likely be non-representative as it would only reflect the most active infants). As such, our sample would experience rapid attrition if other cut-offs were used (e.g., only 74% of participants had trials which each lasted at least 30 s; see Table 2).

**TABLE 2:** Participant attrition in the Lucca et al. (2020) sample using different cut-offs for trial lengths.

Trial time cut-off	% participants retained
20 s	100%
25 s	90%
30 s	74%
35 s	55%
40 s	49%
45 s	41%
50 s	33%
55 s	32%
60 s	27%
65 s	25%
70 s	25%
75 s	23%
80 s	17%
85 s	15%
90 s	13%
95 s	10%
100 s	9%
105 s	8%
110 s	7%
115 s	6%
120 s	4%

*The percentage of participants whose trials each lasted a minimum length rapidly declines after 20 s.*

Finally, infants participated in the recovery trial in order to observe infants’ expectations of task success after the demonstration-test trials. Infants were again faced with a toy in a clear container attached to a rope; this time the apparatus was functional.

### Coding

We focused on select variables coded by Lucca et al. (2020); namely, time spent trying and maximum pulling force. Additionally, data were collected on help-seeking behaviors, and affect. During the recovery trial, the number of hints were recorded.

#### Time Spent Trying

A primary coder watched each participant video and recorded the number of seconds the infant spent trying. An infant was classified as trying if they pulled the rope and looked directly at the toy immediately prior to, during, or after pulling. Behaviors such as swinging the rope side-to-side without making eye contact with the toy, or throwing the rope were classified as off-task behaviors, and therefore, not coded. A secondary coder independently double-coded 100% of the videos, establishing high reliability (*ICC* = 0.95, *p* < 0.001). The trying time data was not normally distributed, therefore the data were square root transformed for analyses of trying time.

#### Maximum Pulling Force

The strength of trying was quantified using a 5 kg S-type load cell discretely connected to the toy. The load cell measured each infant’s pull in pounds per square inch (PSI) and recorded continuous force data on a connected laptop. Maximum PSI was extracted during the first 20 s of each test trial. The force data was not normally distributed, therefore the data were also square root transformed for analyses of force.

#### Help-Seeking Behaviors

A primary coder watched each participant video and tallied the number of help-seeking behaviors displayed during the test trials. Help-seeking behaviors were defined as (1) reaching to the target object, or (2) points toward the target object or experimenter. Since parents were instructed to wear occluding eyeglasses, behavior directed toward the caregiver objectively could not be informative in this task, thus these behaviors were not coded as help-seeking. A secondary coder independently double-coded 100% of the videos, establishing strong reliability (*ICC* = 0.93, *p* < 0.001). For analyses, a composite help-seeking measure was created by summing both reaching and pointing behavior which occurred during each trial.

#### Affect

Participant affect was coded during bouts of trying, using still frames sampled at every 15 frames (i.e., every 510 ms) during trying time. Coders watched close-up recording on each participant’s face and coded emotional reactions using a coding scheme adapted from Repacholi et al. (2016). Coding of positive and negative affect was performed separately, therefore in very rare instances infants could be coded as displaying both negative and positive affect during a single frame. Positive affect was coded if infants displayed characteristic features of a smile (e.g., upturning of the mouth, cheek elevation, raised brows). A secondary coder independently double-coded 50% of the videos for positive affect, establishing high reliability (*ICC* = 0.97, *p* < 0.001). Negative affect was coded if infants displayed characteristic features of frustration or disgust (e.g., down turning of the mouth, furrowed brows, wrinkled nose). A secondary coder independently double-coded 50% of the videos for negative affect, establishing high reliability (*ICC* = 0.96, *p* < 0.001). The total number of frames during a trial that the infant displayed negative affect was used in analyses.

#### Number of Hints Needed During Recovery Trial

The number of hints the infant required from the experimenter to complete the task during the recovery trial were coded. Unlike test trials, task success was possible during recovery. Therefore, hints required was used as a proxy to assess infants’ expectations of task success (i.e., more hints would relate to greater expectations of failure). The number of hints provided by the experimenter was strictly a function of the amount of time passed, as hints were provided at fixed intervals if infants did not solve the task independently. Thus, hints did not reflect the infants’ behavior or help-seeking. A secondary coder independently double-coded 50% of the videos for hints required during recovery, establishing high reliability (*ICC* = 0.96, *p* < 0.001).

### DeepLabCut Coding and Processing

As with our other variables, participant videos were trimmed to the first 20 s of each trial to match the hand-coding scheme employed by Lucca et al. (2020) and to ensure trials had equal data. However, given the variability in trial lengths, we also controlled for the trial length in our models to account for differences in overall time trying between participants. To this end, a human coder classified the length of trials to the nearest second. A secondary coder independently double-coded 25% of the videos for trial length, establishing high reliability (*ICC* = 0.98, *p* < 0.001).

In order to quantify infants’ motion through space, DeepLabCut (DLC), a markerless pose estimation software, was applied to generate data on the rope coordinates for each participant in the *x*- and *y*-axes. Data were not collected in the *z*-axis as this is where pulling by the experimenter model occurred; as such, motion in the *z*-axis was considered imitative, rather than exploratory. The coordinates in the *x*- and *y*-axes were measured in units of pixels and represented the displacement of the rope relative to the origin (i.e., the top left corner of the camera view). Coordinates were generated at a rate of one coordinate pair per video frame (i.e., 30 times per second).

**FIGURE 1:**
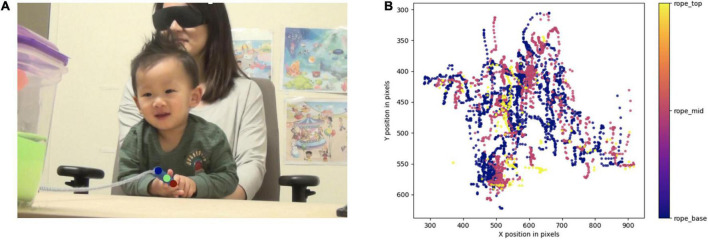
**(A)** A still from a participant video showing markers generated by DLC after training the neural network. The three parts of the rope labeled and tracked were: the base of the rope handle (blue), the middle of the rope handle (green), and the end of the rope handle (red). **(B)** A graphical representation of the coordinates extracted from DLC. Additionally, we have uploaded a video of one of this participant’s test trials with computed marker overlay. Video playback is in real time and can be found here: https://osf.io/5z74k/.

Our goal was to train an artificial neural network (ANN) to identify and track the rope handle through space. The first step was to hand-label frames with our points of interest to create training and test data sets that would be used to train the ANN. Estimation accuracy of a network is improved when trained to track more than one point (Mathis et al., 2018). As such, we trained the network to track three points on the rope: the beginning of the rope handle, the middle of the rope handle, and the end of the rope handle (see Figure 1). However, analyses were conducted using the beginning of the handle, where it attached to the rope. After observing labeled participant videos, this point was the least likely of the three to be occluded by the infants’ hands, and thus, was the most reliable. DLC boasts < 5-pixel error when trained on 100 hand-labeled frames (Nath et al., 2019). In order to minimize the pixel error on our data set, we labeled 200 frames from 10 participant videos. We chose 10 participant videos that represented the diversity of the sample both in terms participant demographics (e.g., participant race, participant gender), and in terms of perceptual features (e.g., shirt color) to ensure the training could be applied to the versatile range of participants present in Lucca et al.’s (2020) sample. Then, 200 frames that featured infants in a variety of positions were manually selected from the 10 participant videos.

Using the recommended neural network, ResNet-50 (Nath et al., 2019), we trained the network on 200,000 iterations of the training set. The trained network had a mean training error of 2.01 pixels, and a mean test error of 3.07 pixels (less than a quarter of a cm). We found this mean error size suitable for our work, thus we did not generate additional iterations of training and the remainder of the participant videos were analyzed using this network. For a detailed user guide, including instructions on creating a training data set, training the network, and evaluating the trained network, please see Nath et al. (2019).

### Assessing DeepLabCut’s Precision

To assess the precision of DLC’s labeling capacity, we had two human coders evaluate the labels generated by DLC. We randomly selected 25% of participants and then randomly selected 20 frames from each participant for evaluation (as 20 frames per participant were used in our training set). We found that on 90% of frames, DLC reported that it had detected the rope handle accurately; on 97% of those frames, human coders agreed that it was correctly labeled. For comparison, Sturman et al. (2020) found DLC was 86 ± 3% accurate compared to human annotated frames, and this outperformed commercial solutions.

### Assessing DeepLabCut’s Validity

Once data was extracted from DLC, it was utilized to construct several measures of exploration (see “Results” section). Before conducting analyses, we wanted to verify that our measures of exploration were not merely a reflection of low-level motor phenomena. To this end, behavioral coding was performed by human raters to identify times when infants were engaged in playing and unproductive movements (i.e., times when the rope was being moved but was not taut). Because infant’s attempts were generally stochastic, shifting rapidly from one behavior to another, coding was performed on the level of 5-s intervals to allow for consistent classification between human raters. Two coders were assigned approximately half the sample each and marked how many intervals displayed unproductive movement in each trial for each participant. Thus, infants could score up to four intervals as unproductive per trial. In addition to coding assignments, approximately 25% of data was double coded and interrater reliability was moderately high (*ICC* = 0.83, *p* < 0.001).

To understand whether our measures of exploration inadvertently captured incidental movement, rather than concerted trying, we performed Pearson correlations between our trial-level measures of imitative similarity and variability with the number of intervals engaged in unproductive movements. Indeed, we did not observe a correlation between either average imitative similarity (*r* = −0.03, *p* = 0.57) or overall variability (*r* = −0.02, *p* = 0.68) and our human-generated measure of unproductive movement. Thus, we did not find evidence that our measures of exploration reflected off-task behaviors or play. This verification provided us with increased confidence of the construct validity of our measures of similarity and variability as exploratory problem-solving strategies.

## Results

### Effects of Firsthand Experience and Model Success on Infants’ Imitative Similarity

In all the demonstrations, infants witnessed the experimenter modeling straight back pulling of the rope. Our first goal was to understand whether infants’ trying attempts were similar to the demonstration of the experimenter or whether infants altered the angle of their pulling along the *x-* and *y-*axes. To this end, we produced measures to capture divergence from imitation by centering participants’ raw displacement values in each axis relative to imitative (i.e., straight back) pulling. As not all participants engaged in imitative pulling, we were unable to center each participant’s attempts relative to their own imitation. However, data from all participants who engaged in imitative pulling (*n* = 78) were utilized to generate average imitative estimates. A given pull was defined as imitative if the rope handle did not go beyond the shoulders in either axis, and if the infant was properly seated in their parents’ lap (i.e., not straining or bouncing). These video clips were run through the neural network to obtain an average *x-*value (458.29 pixels; 35.25 cm from the left of the camera frame) and an average *y-*value (347.79 pixels; 26.75 cm from the top of the camera frame) for imitative pulling. These values were subtracted from infants’ raw displacement values for each axis. Because we valued divergence from imitation in both directions (i.e., right and left, up and down), the absolute value of each deviation value was then taken.

Once these values were calculated, we performed two one-sample *t*-tests to compare the displacement values in each axis to imitation (i.e., 0) to understand whether infants’ pulling attempts differed significantly from the experimenter. To make use of the rich data produced by DLC, each *t*-test considered 60 points per participant (20 coordinate pairs per trial for each of the three trials), excluding outliers.^1^ In each of the conditions, infants experienced failure once they attempted to solve the means-end problem on their own. Thus, we expected that infants as a group would generate new strategies to improve upon the strategy modeled by the experimenter. Pulling attempts in both the *x-*axis [*M* = 191.01 pixels/14.69 cm, *SE* = 1.63 pixels, *t*(5670) = 117.09, *p* < 0.001] and the *y-*axis [*M* = 154.08 pixels/11.85 cm, *SE* = 1.16 pixels, *t*(5751) = 133.30, *p* < 0.001] differed significantly from imitation. Thus, infants’ attempts differed significantly from the experimenter in each axis. We also sought to understand whether pulling attempts differed between the two axes, thus we additionally performed a paired-sample *t*-test to understand whether there was greater deviation in one axis than the other. Indeed, infants’ pulling attempts in the *x-*axis deviated from imitation to a significantly greater extent than in the *y*-axis [*M*_diff_ = 37.36 pixels/2.87 cm, *t*(5669) = 18.67, *p* < 0.001]. Thus, infants did not merely replicate the actions of the experimenter and their attempts appeared to differ to a greater extent in the *x-*axis than the *y-*axis.

**FIGURE 2:**
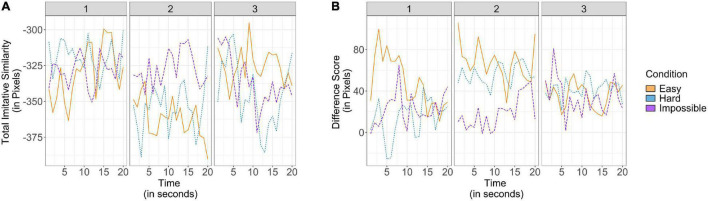
Imitative similarity plotted as **(A)** an additive score combining variation from imitation in the *x*- and *y*-axes and **(B)** a difference score subtracting variation from imitation in the *y*-axis from the *x*-axis. Each graph is plotted by time, divided by condition and trial number. In the case of the additive score, greater values represent greater imitation, while in the case of the difference score, greater values represent greater variation in the *x*-axis relative to the *y*-axis.

Our next goal was to understand how infants’ imitative similarity was influenced by the effort and success of the adult model and firsthand experience with failure. To this end, two measures were constructed using the absolute values from the *x-* and *y-*axes: (1) a difference score to allow us to understand if infants systematically varied the axis of their exploration, made by subtracting values in the *y-*axis from the *x-*axis, and (2) an additive score representing overall imitative similarity by summing, then reverse-scoring, the scores for interpretability. Thus, for the difference score, positive values indicate greater deviation from imitation in the *x-*axis than the *y-*axis, and for the imitative similarity measure, a score of 0 indicated imitative pulling in both axes and greater negative values represent greater exploration (see Figure 2). Once these measures were calculated, linear mixed effects models were built to predict changes in the two measures, respectively. In each model, participants were entered as random effects and the main effects of condition, trial number, and time within the trial (in seconds) were entered as predictors. Further, individual variation in overall trial length and motor skill may lead to differences in infants’ experiences trying in this task. Therefore, we also entered the main effects of overall trial length and motor skill as covariates to control for these effects. As we expected that greater experience with failure (both within trials and across trials) would decrease the utility of imitation, we additionally checked for an interaction between trial number and time within the trial. Finally, as condition was a categorical variable with three categories, we used the Easy condition as a baseline in accordance with Lucca et al. (2020), though it is worth noting that the pattern of results is the same regardless of specified baseline condition.

In the case of our difference score, we did not expect to find systematic variability as there was no information provided by the experimenter or across time which would suggest exploration in one axis would be more effective than in the other. Indeed, there were no significant main effects of condition, time within trial, nor an interaction between trial number and time (all *p’*s > 0.39). However, there was an effect of trial such that infants’ pulling attempts differed from imitation to a greater extent in the *x-*axis than the *y-*axis in later trials [*t*(5602) = 2.27, *p* = 0.02, β = 8.89, *SE* = 3.92]. Thus, infants appeared to explore locations that were more disparate from the experimenter particularly in the *x-*axis across trials. This result may be due to the physical limitations of the study design, wherein infants sat in their caregiver’s lap and were less able to move vertically than horizontally. Critically, there was not a significant effect of motor skill on the difference score (*p* = 0.96).

On the other hand, we thought that our measure of imitative similarity could be sensitive to the effort and success of the adult model, as infants received varying information about the success of the modeled solution, and to firsthand evidence, as failure would suggest a necessity for strategy diversification. Our model of imitative similarity revealed a significant main effect of trial such that infants’ attempts became more similar to the experimenter over trials [*t*(5497) = 2.14, *p* = 0.03, β = 8.09, *SE* = 3.78] and a main effect of time such that infants’ attempts became more imitative as trials progressed [*t*(5475) = 2.51, *p* = 0.01, β = 1.63, *SE* = 0.65], as well as a significant interaction between trial number and time such that on later trials, infants pulling attempts diverged more from imitation over time [*t*(5475) = −3.08, *p* = 0.002, β = −0.93, *SE* = 0.30]. As before, we did not observe an effect of motor skill in our model of imitative similarity (*p* = 0.22). It is worth noting that the effects in this model were relatively small, and that our prior analyses revealed infants’ pulling attempts were overall significantly different from the experimenter in both axes. Thus, though infants’ pulling attempts became more imitative over time, these attempts were still overall dissimilar to the experimenter. Finally, we did not observe any effects of condition on imitative similarity (both *p*’s > 0.78). Thus, imitative similarity seemed to respond more to information gained through firsthand experience than from the adult model.

### Effects of Firsthand Experience and Model Effort and Success on Infants’ Variability

**FIGURE 3:**
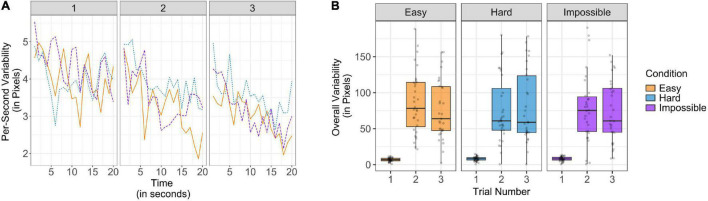
Variability plotted **(A)** using a per-second score to represent variability in real-time and **(B)** using an overall score to represent the variability created across the entire trial, divided by condition and trial number. For each score, greater values represent greater variability and exploration.

Our analyses of imitative similarity allowed us to understand whether the locations of infants’ pulling attempts varied significantly from the experimenter and how they varied over time. However, in the face of continued failure, it is both sensible to divest from imitation and also to test multiple locations and solutions as each new attempt fails. Thus, our next goal was to complement our understanding of exploration by evaluating the variability in infants’ pulling attempts. To index spatial variability, the coordinates returned from DLC for each participant and each trial were used to calculate standard deviations of displacement in both axes. Because standard deviation is highly dependent on the mean of a given time interval, we calculated two variability scores: (1) a per-second variability score, representing the average standard deviation of movement in the *x-* and *y-*axes during the previous second, which responded to the local means of displacement in the previous second, and (2) an overall variability score, representing the average standard deviations of displacement in the *x-* and *y-*axes during the trial, which responded to the global means of displacement over the entire trial (see Figure 3). Because of skew in the per-second variability score and for consistency between measures, the measures used in analyses were square root transformed.

To understand how per-second variability was affected by the effort and success of the adult model, and firsthand experience with failure, a mixed effects model with the same specifications as the previous models was constructed. Participants were entered as random effects and the main effects of condition, trial number, and time within the trial (in seconds) were entered as predictors, the main effects of trial length and motor skill as covariates, as well as the interaction between trial number and time. As before, we expected that the varying success of the adult model between conditions could have an effect on variability. Likewise, we expected that firsthand evidence could have an effect, though we did not have specific hypotheses as to whether per-second variability would decrease, as failure may encourage lesser overall engagement, or increase, as failure may also potentiate exploration. This model revealed only trending effects of time such that per-second variability decreased over time [*t*(5479) = −1.84, *p* = 0.07, β = −0.03, *SE* = 0.02], and a trending interaction between trial and time [*t*(5479) = −1.89, *p* = 0.06, β = −0.02, *SE* = 0.01], such that per-second variability decreased to a greater extent on later trials. There were no effects of condition nor trial number (all *p’*s > 0.24). Likewise, we did not observe an effect of motor skill on per-second variability (*p* = 0.35). Thus, as infants experienced greater firsthand failure, they exhibited less real-time variability, but we did not find evidence of an effect of the effort and success of the adult model.

On the other hand, to understand how overall variability varied as a function of condition and trial number, a linear mixed effects model was built to predict overall variability. In this case, we hypothesized that overall variability should increase across trials as greater firsthand experience with failure should suggest that previously tested solutions would not succeed. Participants were entered as random effects and the main effects of trial number and condition were entered as predictors. We also entered the main effects of trial length and motor skill into the model as covariates. As before, we used the Easy condition as a baseline, though it is worth noting the pattern of results was the same regardless of baseline. We found a main effect of trial number, such that spatial variability increased across trials [*t*(222) = 13.35, *p* < 0.001, β = 2.71, *SE* = 0.20], but there were no significant effects of condition (both *p’*s > 0.43). Therefore, while infants’ spatial variability responded to firsthand failure across trials, we did not find evidence that it was also sensitive to the effort and success of the adult model. As in our other models, motor skill did not have a significant effect on overall variability (*p* = 0.74). This analysis of overall variability revealed a markedly different pattern than our analysis of per-second variability. We discuss the potential explanations and implications of these results in the Section “Discussion.”

### Predicting Individual Differences in Exploration

Our second analytic goal was to understand how individual differences in performance measures predicted infants’ exploration, in order to better understand the processes that lead to exploration. The distributions of many of our performance measures exhibited substantial positive skew. While we transformed these variables as necessary to reduce skewness (e.g., trying time, maximum force, overall variability), we additionally employed 20% percentage-bend correlations to increase the robustness of analyses predicting exploration utilizing negative affect, maximum pulling force, help-seeking, and trying time, respectively. Pearson correlations may lack robustness with this type of data, as small shifts in marginal distributions or outliers can lead to substantial variations in correlation estimates (Wilcox, 1994). Thus, utilizing 20% percentage-bend correlations allowed our analyses to have greater robustness against the skew exhibited in our performance measures. We conceptually treated each performance measure as a predictor of imitative similarity and the square root of overall variability, respectively. However, as we did not have specific hypotheses about the direction of the effects, these analyses were all exploratory and correlational.

**FIGURE 4:**
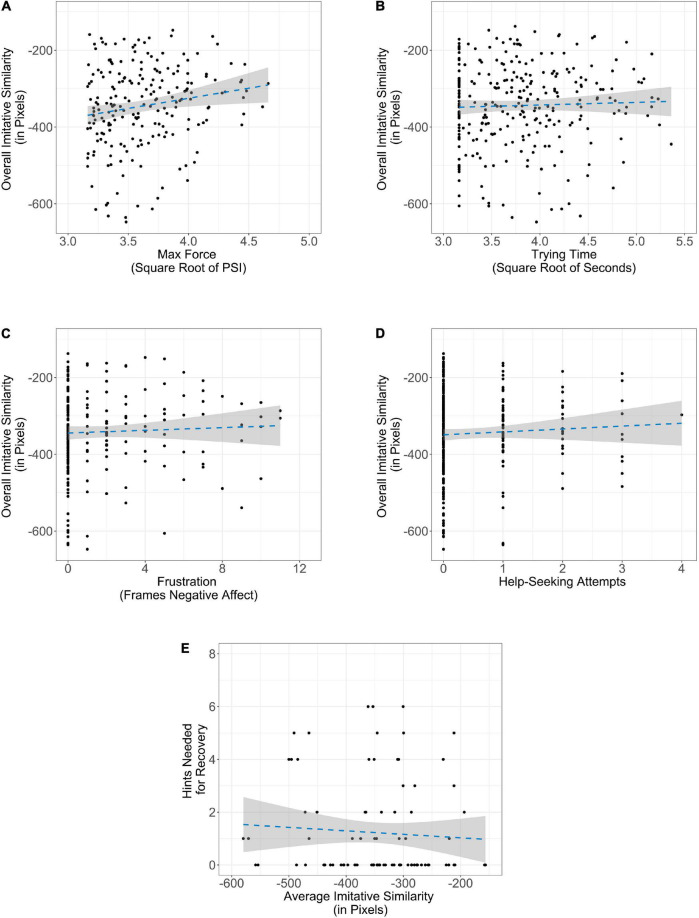
Relationship between performance measures and average imitative similarity: **(A)** maximum pulling force, **(B)** trying time, **(C)** negative affect, **(D)** help-seeking, and **(E)** hints during recovery. The shaded region along the line of best fit represents standard error.

**FIGURE 5:**
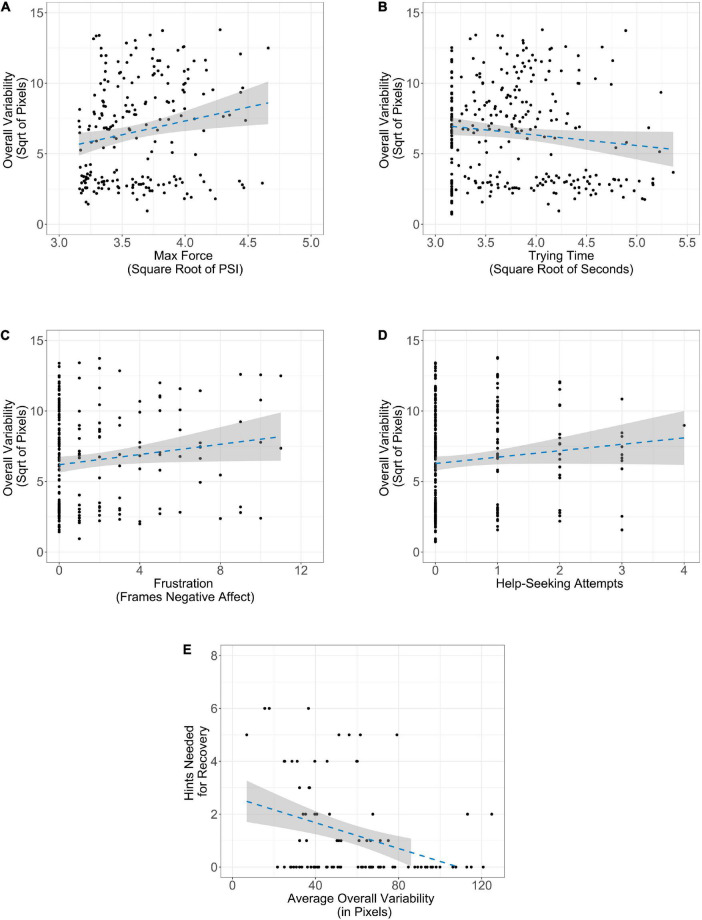
Relationship between performance measures and overall spatial variability: **(A)** maximum pulling force, **(B)** trying time, **(C)** negative affect, **(D)** help-seeking, and **(E)** hints during recovery. The shaded region along the line of best fit represents standard error.

We first looked to see how the performance measures related to imitative similarity. Increases in maximum pulling force were related to decreases in imitative similarity (ρ*_*pb*_* = −0.16, *p* = 0.03). Thus, infants who utilized greater maximum pulling force tended to diverge more from imitation. However, there were no other trending or significant relationships observed between imitative similarity and the performance measures (all *p’*s > 0.28; see Figure 4). We next performed individual difference analyses of spatial variability (see Figure 5). Regarding force, we found that increases in maximum pulling force were associated with greater overall variability (ρ*_*pb*_* = 0.22, *p* = 0.003). Therefore, infants who utilized greater maximum pulling force tended to generate greater spatial variability. We also found that increases in trying time were associated with lower spatial variability (ρ*_*pb*_* = −0.27, *p* < 0.001). Lastly, there was a trending relationship with affect and variability. We found that increased negative affect tended to be associated with greater overall variability (ρ*_*pb*_* = 0.13, *p* = 0.08). Thus, infants who were more frustrated may have generated greater spatial variability. Finally, we did not find evidence of a relationship between help-seeking and overall variability (ρ*_*pb*_* = 0.11, *p* = 0.13).

### Predicting Differences in Expectations of Task Success

Finally, we conducted 20% percentage-bend correlations to investigate the relationship between expectations of task success (i.e., the number of hints infants needed on the recovery trial) and imitative similarity and spatial variability, respectively. We hypothesized that infants who explored more in preceding test trials would require less support during the new iteration of the task, as each new strategy employed would present a new opportunity for success. Since there was only one measure of hints required during the recovery trial for each participant, we averaged the standard deviation of spatial displacement across the three trials, as well as the additive imitative similarity scores, for analyses. There was not a significant relationship between hints required during the recovery trial and imitative similarity (*p* = 0.38). However, hints required and overall variability were moderately, negatively correlated (ρ*_*pb*_* = −0.37, *p* < 0.001) such that infants who had higher average overall variability during the test trials required fewer hints during the recovery trials. Importantly, our measures of spatial variability and hints required during the recovery trial were independently collected. Since spatial variability was measured prior to the recovery trial, it seems that spatial variability while problem-solving predicted the number of hints required in the recovery trial.

## Discussion

### Insights Gained About Infants’ Problem-Solving Strategies

This paper’s primary conceptual objective was to investigate the influence of the effort and success of an adult model, and firsthand experience with failure on infants’ problem-solving approaches by quantifying the extent to which these attempts deviated from modeled solutions. To this end, we considered multifaceted components of infants’ problem-solving approaches by applying DLC to generate objective, high-quality data: imitative similarity and spatial variability. Our findings revealed that these exploratory facets of problem-solving were relatively immune to social input (i.e., the effort and success of an adult model) but responded to firsthand failure across and within trials. Thus, although imitative similarity and spatial variability were influenced by some of the same factors that influence the time spent problem-solving (see Lucca et al., 2020), focusing on these new measures of exploration yielded new information about the nature of infants’ problem-solving approaches.

By investigating imitative similarity, we were able to assess the extent to which infants’ pulling behaviors deviated from the modeled solution. This process revealed that infants’ pulling behaviors were significantly different from the experimenter model they observed. Thus, when given an unsolvable task, infants as a group generated solutions which were unique from the experimenter by testing new locations. Imitative similarity also varied across time; within trials, infants’ approaches tended to become slightly more imitative as they experienced greater failure, except on later trials, wherein their approaches became less imitative over time. In this task, exploration is useful in that the experimenter’s solution demonstrably fails when attempted. In early trials, infants may test different solutions then converge toward the experimenter’s solution once they experience failure with exploration. On the other hand, on later trials, infants may increase exploration after repeated experience failing using the modeled solution. These results suggest that infants’ problem-solving approaches respond to firsthand experience with failure.

Likewise, we were able to investigate the variety exhibited in infants’ problem-solving approaches by measuring variability, rather than just overall similarity. Our spatial variability findings differed depending on the timescale utilized for analysis. While per-second variability decreased both within and between trials, overall variability actually *increased* between trials. These results can be interpreted as complementary, as standard deviation is highly dependent upon the mean of a given time interval. As such, if infants spent several seconds testing a given location or strategy (i.e., high similarity to the mean of each second), but also tested several disparate locations across the entire trial (i.e., lower similarity to the overall mean), they would demonstrate low per-second variability but high overall variability. Thus, the explanation most consistent with our collection of findings may be that infants persisted for longer in each location they tested as they experienced greater firsthand failure but tested a wider variety of locations throughout the entirety of the trials. Subsequently, spatial variability during test trials predicted recovery trial performance. Infants who produced greater variability during test trials received fewer hints during recovery, requiring less support in the new iteration of the task. Thus, it seems that spatial variability predicted support needed during later recovery trials, suggesting that children who explored more had higher expectations of task success.

Of course, alternate explanations could exist for these results. Most concerningly, our measures of imitative similarity and variability could merely reflect incoordination. However, we find this interpretation quite unlikely because we controlled for motor skill within each of our models. If indeed our measures were merely a reflection of a lack of coordination, we would expect that lower motor skill would be related to each measure. However, we did not observe any significant effects of motor skill on imitative similarity or spatial variability. Likewise, our human coding of unproductive movement was not correlated with imitative similarity or variability. In light of these findings, we find the most consistent explanation is that our results reflect nuanced adjustments to problem-solving approach as infants experienced failure. These adjustments may be deliberate or implicit but are observable in infants’ behavior regardless.

While these results display similarities to Lucca et al. (2020), they also indicate departures. In the original study, infants’ trying time responded both to the effort and success of the adult model, and to firsthand experience with failure. Thus, both studies indicated an effect of firsthand experience such that infants’ problem-solving approaches changed with increased failure, but we did not find evidence for an effect of social input. This is surprising given that similar work also suggests that infants infer appropriate strategies based on social input (e.g., Gweon and Schulz, 2011) and that the demonstrations provided different information about the solution’s efficacy. For example, while the Impossible condition cues that the modeled solution is ineffective, the Hard condition cues that the modeled solution will work eventually. As such, our results point to a potential disassociation between the duration of problem-solving and the approach adopted during problem-solving. It may be the case that exploration represents a more implicit component of problem-solving and responds to firsthand evidence (i.e., failure) but does not become consciously integrated across domains like trying time.

### Utility of Applying DeepLabCut

Importantly, this project also sought to illustrate the feasibility and utility of implementing DLC in the analysis of archival data. By identifying a motoric proxy for a cognitive phenomenon (i.e., exploration) we were able to apply computer vision *post hoc* to a previously collected data set to reveal novel insights about problem-solving. This case study provides one example of DLC’s application, but the fine-grained data that DLC produces could also be used in more sophisticated computational models and statistical techniques, much like linguistic corpora have been utilized (e.g., Redington et al., 1998; Yang, 2013; Meylan et al., 2017; Bergey et al., 2021). Importantly, DLC is particularly useful when in-person data collection is impossible. DLC can utilize archival data which is an invaluable tool (Gordon et al., 2015), and gives researchers access to high-quality or even rare data (e.g., data from samples which are not Western, Educated, Industrialized, Rich, and Democratic; see Rad et al., 2018; Syed et al., 2018). However, archival data is often collected to answer specific questions and, consequently, the stimulus design may not easily lend itself to new questions. In these cases, DLC provides researchers with open-access tools to answer additional questions that are otherwise very difficult or time-consuming for human coding, extending the lifecycle of existing archival data as in the case of our data. Thus, the advantages of DLC are pertinent for archival research both when in-person data collection is and is not possible.

### Implications for Theories of Problem-Solving and Related Phenomena

Classic work demonstrates that young infants have perseverative tendencies, wherein they will continue to apply previously successful solutions to solve problems even when they are no longer effective. Infants’ A-not-B task performance classically illustrates this phenomenon: after a 10-s delay, even 12-month-olds demonstrated perseverative errors by continuing to search in the original location an object was hidden instead of its current location (Diamond, 1985). Although perseveration on this particular task diminishes across the second year of life (Lockman and Pick, 1984; McKenzie and Bigelow, 1986; Aguiar and Baillargeon, 1999), perseveration more broadly construed persists into at least the preschool years in various motor-based tasks (Schutte and Spencer, 2002; Mash et al., 2003; Sharon and DeLoache, 2003; DeLoache et al., 2004; Smitsman and Cox, 2008; Schmuckler, 2013). In contrast to these findings, within the context of our study, 18-month-olds demonstrated relative flexibility, testing solutions unique from the adult model, testing a greater variety of solutions across trials, and varying imitative similarity based on trial number. These findings suggest that perseverative tendencies may vary both with the nature of the task and infants’ experiences during the task. Overall, our study juxtaposes previous work on perseveration by showcasing infants’ ability to generate productive responses in the face of failure.

Tendencies toward imitation versus exploration can also be understood through the explore-exploit tradeoff, a common framework describing the inherent tension between exploiting known solutions for rewards and taking time to explore better solutions (Mehlhorn et al., 2015). Within this framework, imitation can be understood as exploitation, as infants can conserve mental resources while gaining the benefits of a known solution. Conversely, exploration may produce better solutions but may come at the expense of efficiency, as generating new solutions requires trial-and-error. Longitudinal comparisons have revealed that children tend to explore to a greater extent than adults, choosing to gather information rather than rely on exploiting known effects, with this tendency reducing over development (Gopnik et al., 2015, 2017; Sumner et al., 2019; Gopnik, 2020). However, this research has generally been done with children who are preschool-age or older, due to the cognitive demands of the tasks employed. Our findings demonstrate a ready tendency to explore novel solutions in infants, suggesting that this tendency may be present from an even younger age. Future work could adopt similar paradigms to allow for a full developmental comparison beginning in infancy.

Previous work from this perspective has also differentiated exploration into two subsections: directed and random exploration (Meder et al., 2021; Wilson et al., 2021). Whereas directed exploration serves to sample the areas of greatest uncertainty in a problem space, random exploration simply generates variability. Importantly, both forms of exploration are posed as adaptive, as directed exploration allows for the inspection of features which are likely to produce rewards, but random exploration allows for the discovery of less obvious features which may also be useful. Critically, our methodology did not differentiate between directed and random exploration, as deviation and variability could represent both random, implicit micro-explorations and qualitatively distinct strategies. Thus, future work may adopt methods that elucidate this distinction.

Our findings regarding exploration may also be suited to a larger literature characterizing children’s intuitions about effort exertion and problem solving as fundamentally rational. Effort is costly, requiring metabolic resources and creating inherent opportunity costs. As such, children as young as 6 months old expect others to utilize the most efficient paths possible to obtain their goals (Brandone and Wellman, 2009; Scott and Baillargeon, 2013; Skerry et al., 2013; Liu and Spelke, 2017). The naïve utility calculus integrates these intuitions into a framework explaining how children take advantage of the utility of others’ actions to infer a wide variety of information including desires, preferences, and prosocial tendencies (Jara-Ettinger et al., 2015a,b, 2016). Recent research has begun to elaborate how children also display effort efficiency in their own actions (Leonard et al., 2020; Lucca et al., 2020; Rett and Walker, 2020). The results presented here are consistent with this larger framework, demonstrating that infants engaged in several exploratory behaviors which increased the utility of their actions. Infants did not merely copy the approach of the experimenter when they did not experience success, but rather deviated from the demonstration. As infants were confronted with their own failure, they also generally increased their exploration, increasing their deviation from imitation and trying a greater number of exploratory strategies. In other words, infants’ approaches responded to information about the productivity of imitation, as well as the productivity of each strategy that they employed. Further, greater exploration related to greater expectations of task success. As infants tested new methods, their expectations of success may have been buffered through failure as there is a possibility that each untested strategy could lead to success. While these results are still speculative, they suggest that infants may engage with problems in nuanced ways to maximize probabilities of success rather than merely giving up or perseverating.

### Limitations

The primary limitation of this study is the correlational nature of our data. As with any archival research, if the questions under investigation pertain to variables that were not directly manipulated in the original study, researchers are limited in making causal assertions about their data. In the case of our study, we were only able to make inferential claims about variables which were collected or manipulated independently (i.e., adult modeling, trial number, and recovery trial performance) but further work will be required to make definitive conclusions about the relations observed between other variables, particularly the role of exploration within a larger motivational framework as described in our individual difference analyses. However, correlational work serves as an important exploratory space for generating new research questions and as such, the efficiency of DLC makes it an ideal option for researchers who are endeavoring to test the feasibility or theoretical validity of a research question before investing the time designing an appropriate paradigm, collecting data, and processing data (either in-person, or online).

## Future Directions and Conclusion

This work provides rich theoretical grounds for future research through its correlational findings. In addition to the directions identified above, we would recommend a further investigation of other facets of exploration and the developmental trajectories of exploratory tendencies. Here, we considered two potential facets of exploration, demonstrating that these two facets responded to firsthand experience with failure. Future work should also consider which other facets may comprise a full constellation of exploratory problem-solving behavior beyond imitative similarity and variability. Likewise, if the measures elaborated in this paper reflect exploration, they raise further questions about the developmental trajectory of these abilities (Muentener et al., 2018). Finally, our correlational analyses of individual differences in spatial variability and other facets of performance raise productive possibilities for empirical investigation and replication. Exploration may be one component of an interplay between failure and problem-solving. As such, it may explain divergence between learners, in which failure results in divestment for some but growth and learning for others. This interpretation is corroborated by our recovery results, which suggest that greater exploration during problem-solving leads to greater expectations of task success. Perhaps encouraging children who do not naturally produce large exploratory variability to explore will buffer motivational losses.

## Data Availability Statement

The original contributions presented in the study are publicly available. These data can be found here: https://osf.io/sydqz/.

## Ethics Statement

Written informed consent was obtained from the individual(s), and minor(s)’ legal guardian/next of kin, for the publication of any potentially identifiable images or data included in this article.

## Author Contributions

HS, MR, and JS made substantial contributions to the conception and design of the work. HS implemented DeepLabCut and coordinated data processing. MR performed all data analyses. MR and JS provided critical oversight and feedback of the work. HS and MR wrote the manuscript. JS provided critical feedback on the manuscript. All authors contributed to the article and approved the submitted version.

## Conflict of Interest

The authors declare that the research was conducted in the absence of any commercial or financial relationships that could be construed as a potential conflict of interest.

## Publisher’s Note

All claims expressed in this article are solely those of the authors and do not necessarily represent those of their affiliated organizations, or those of the publisher, the editors and the reviewers. Any product that may be evaluated in this article, or claim that may be made by its manufacturer, is not guaranteed or endorsed by the publisher.

## Footnotes

1 In all analyses, outliers which were more than 2.5 SD from the mean were removed using pairwise deletion.
